# Diacron reactive oxygen metabolites and biological antioxidant potential tests for patients with age-related macular degeneration

**DOI:** 10.1186/s12886-020-01334-y

**Published:** 2020-02-18

**Authors:** Toshiyuki Matsuura, Hiroki Kaneko, Kei Takayama, Rei Shibata, Keiko Kataoka, Seina Ito, Taichi Tsunekawa, Hideyuki Shimizu, Ayana Suzumura, Rina Namba, Yasuki Ito, Toyoaki Murohara, Hiroko Terasaki

**Affiliations:** 1 Department of Ophthalmology, Nagoya University Graduate School of Medicine, 65 Tsurumai-cho, Showa-ku, Nagoya, 466-8550 Japan; 2 Department of Ophthalmology, National Defense Medical College, Saitama, Japan; 3 Department of Advanced Cardiovascular Therapeutics, Nagoya University Graduate School of Medicine, Nagoya, Japan; 4 Department of Cardiology, Nagoya University Graduate School of Medicine, Nagoya, Japan

**Keywords:** Age-related macular degeneration, Diacron reactive oxygen metabolites, Biological antioxidant potential, Oxidative stress, Choroidal neovascularization

## Abstract

**Background:**

Previously, we showed that serum malondialdehyde (MDA) was significantly higher in patients with neovascular age-related macular degeneration (nAMD) than in those without AMD. The Diacron reactive oxygen metabolites (d-ROMs) and biological antioxidant potential (BAP) tests are known markers of oxidative stress. The aim of this study was to use d-ROMs and BAP tests to evaluate changes in systemic oxidative stress in patients with nAMD.

**Methods:**

Blood serum samples were collected from 34 patients with nAMD (mean age: 76.5 ± 7.7 years; 22 men) and 20 control subjects (mean age: 62.9 ± 14.0 years; 10 men), and d-ROMs and BAP tests were examined.

**Results:**

In men, the mean level of d-ROMs for the nAMD patients was significantly higher than that for the controls (312.0 ± 52.4 vs. 275.1 ± 45.5 U.CARR, respectively; *P* < .05). There was a significant correlation between d-ROM level and CNV lesion area in the male nAMD group (*r* = .42, *P* = .05). There were no significant differences in mean BAP test results between the nAMD patients and controls for either sex (men: 2241 ± 549 vs. 2136 ± 246 μmol/L; women: 2263 ± 292 vs. 2335 ± 161 μmol/L).

**Conclusion:**

The d-ROMs test may provide a useful indicator of nAMD in men but not in women.

## Background

Age-related macular degeneration (AMD) is a leading cause of blindness, and the increasing number of AMD patients has had a severe economic impact in many developed countries [1, 2]. Advanced AMD is classified according to its pathophysiology as either neovascular AMD (nAMD) or geographic atrophy (GA) [3]. nAMD is characterized by choroidal neovascularization (CNV) and an increase in intraretinal/subretinal fluid, which is strongly associated with the overexpression of vascular endothelial growth factor (VEGF). Multiple risk factors have been shown to contribute to the pathogenesis of nAMD, including obesity [4], hypertension [5], smoking [6], and light exposure [7, 8]; it has been presumed that these act through inducing oxidative stress [9–11]. Previously, we showed that serum malondialdehyde (MDA), a marker of oxidative stress, was significantly higher in patients with nAMD than in those without AMD, and that there was a significant correlation between serum MDA levels and the area of CNV [12].

The Diacron reactive oxygen metabolites (d-ROMs) test for measuring reactive oxygen metabolites (ROMs) in blood has been used for various biological and medical purposes [13, 14]. This test uses the Free Radical Analytical System (Diacron International, Grosseto, Italy) [15–17]. The main component of ROMs is hydroperoxide [16, 17], which causes cell death and tissue damage [18, 19]. Despite its power as an oxidant, hydroperoxide in blood is relatively stable compared to its parent free radicals, and its level can be adequately detected. Use of this analyzer also allows the determination of biological antioxidant potential (BAP) in blood. The BAP test measures the capacity of blood to convert an Fe^3+^ reagent to Fe^2+^ [20, 21]. In combination, the d-ROMs and BAP tests provide a comprehensive evaluation of oxidative stress.

In this study, we hypothesized that the d-ROMs and BAP tests could be useful markers of nAMD. We tested this hypothesis separately in groups of male and female nAMD patients and controls, comparing d-ROM and BAP test results. The purpose of this study was to use the d-ROMs and BAP tests to evaluate changes in systemic oxidative stress, and to investigate the correlation between these markers and clinical condition in patients with nAMD.

## Methods

### AMD patients and control subjects

The study included 34 patients with nAMD (22 men and 12 women, mean age 75.5 ± 7.7 years), and 20 patients in the control group (10 men and 10 women, mean age 67.0 ± 13.1 years). All of the subjects were aged > 50 in nAMD group and > 40 years in control group and their axial length was in the range 23.0–26.0 mm. Patients with polypoidal choroidal vasculopathy, retinal angiomatous proliferation, or myopic CNV were not included in the patient group; only typical nAMD was examined. To avoid factors that may affect the results of the d-ROMs and BAP tests, patients with diabetes, dyslipidemia, hypertension, cerebrovascular disorders, cardiovascular disorders, and cancer, as well as those under extreme stress, were also excluded. The diagnosis of nAMD was established on the basis of age, clinical examination, fundus photography, optical coherence tomography, and fluorescein angiography (FA), as previously described [22, 23]. The control group comprised patients with other ocular diseases, including cataract, retinal detachment, and macular hole, and epiretinal membrane. We conducted this cross-sectional study in accordance with the guidelines of the Declaration of Helsinki; the protocol was registered to the Clinical Trial Registry of UMIN (Registered number UMIN000032946, Registered date: Jun 11th, 2018) and approved by the Nagoya University Hospital Ethics Review Board (Number 2012–0340-4). We obtained written informed consent from all participating patients.

### Best-corrected visual acuity

The subjects’ best-corrected visual acuity (BCVA) was measured using a standard Japanese visual acuity chart. For the statistical analysis, decimal BCVA was converted to the logarithm of the minimum angle of resolution (logMAR).

### FA imaging and evaluation of CNV area

FA was recorded for all patients in the nAMD group using cSLO (Heidelberg Retina Angiograph, HRA2; Heidelberg Engineering, Dossenheim, Germany), as previously described [24–26]. To evaluate the area of the CNV lesion, we traced the border of the area of hyperfluorescein in images captured at 5 min, and counted the pixels using the NAVIS bundled software (Nidek Co. Ltd., Aichi, Japan). The measurements were made by two observers (T.M. and K.T.), both blinded to the subjects’ clinical features. The measurements were converted from number of pixels to area (in mm^2^) on the basis that one pixel in the FA image had an area of .0004 mm^2^.

### The d-ROMs and BAP tests

A 2 mL serum sample was collected from each subject and serums were divided into tubes and stored at − 80 °C until use. They were thawed only once before examination.. The indicators of oxidative stress were measured using the free radical analyzer FREE CARRIO DUO (Diacron International). The serum sample was placed in a cuvette filled with a buffer, and a color-developing chromogen (N,N-diethyl-p-phenylenediamine) was added and mixed in, resulting in the chromogen substrate being oxidized by the free radicals. The color change in the cuvette was measured optically in a photometer and recorded in units of U.CARR. BAP was also measured using the free radical analyzer FREE CARRIO DUO. A chromogen for BAP (a reagent containing trivalent iron; 50 μL) was added to a cuvette and the color-developing concentration was measured using a photometer (with the results recorded in μmol/L).

### Statistical analysis

The data are presented as mean ± the standard error of the mean (SEM). The Mann–Whitney U test was used to compare data between the nAMD and control groups, with separate analyses for the men and women, and Spearman’s correlation was used to evaluate the correlation between d-ROMs and BAP values and age, logMAR BCVA, and CNV area. *P* < .05 was considered statistically significant.

## Results

### Subject characteristics, and serum d-ROM and BAP levels

**Fig. 1:**
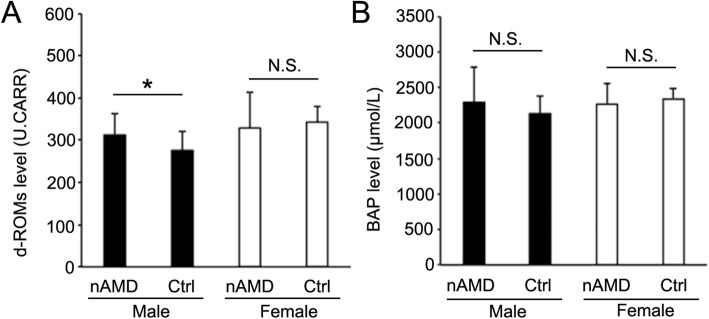
The mean levels of serum d-ROMs (**a**) and BAP (**b**) in male and female patients with neovascular age-related macular degeneration (nAMD) and control subjects (Ctrl). The bars indicate the mean + the standard error of the mean. d-ROMs levels were significantly higher in the male patients with nAMD than in the male control subjects. *P < .05; N.S. = no significant difference. d-ROM = Diacron reactive oxygen metabolite; BAP = biological antioxidant potential

The subjects’ characteristics are presented in Table 1. In men, the mean level of d-ROMs for the nAMD patients was significantly higher than that for the controls (312.0 ± 52.4 vs. 275.1 ± 45.5 U.CARR, respectively; *P* < .05; Fig. 1a). In women, however, there was no significant difference in mean levels of d-ROMs between the nAMD patients and controls (328.6 ± 85.7 vs. 342.5 ± 37.1 U.CARR; *P* = .16; Fig. 1a). There were no significant differences in mean BAP test results between the nAMD patient group and control group for either sex (men: 2241 ± 549 vs. 2136 ± 246 μmol/L, *P* = .57; women: 2263 ± 292 vs. 2335 ± 161 μmol/L, *P* = .43) (Fig. 1b). There was no significant difference between the male and female nAMD patients in the mean area of CNV lesions (3.05 ± 2.36 mm^2^ and 3.15 ± 3.23 mm^2^, respectively; *P* = .80).

**Table 1:** Characteristics of the subjects in the nAMD and control groups

	nAMD		Control	
	Male	Female	Male	Female
Number	22	12	10	10
Age (years)	73.9 ± 7.65	78.5 ± 7.05	66.7 ± 10.5	67.3 ± 15.9
logMAR BCVA	0.44 ± 0.34	0.55 ± 0.45	0.23 ± 0.41	0.21 ± 0.26
d-ROM (U.CARR)	312 ± 52.4	329 ± 85.7	275 ± 45.5	343 ± 37.1
BAP (μmol/L)	2286 ± 500	2263 ± 292	2136 ± 246	2335 ± 161
Current smoker (%)	22.7	8.3	20.0	10.0

Data are mean ± SEM

*nAMD* neovascular age-related macular degeneration, *logMAR* logarithm of the minimum angle of resolution, *BCVA* best corrected visual acuity, *d-ROM* Diacron reactive oxygen metabolite, *BAP* biological antioxidant potential

### Correlations between serum d-ROMs levels and nAMD patient characteristics

**Fig. 2:**
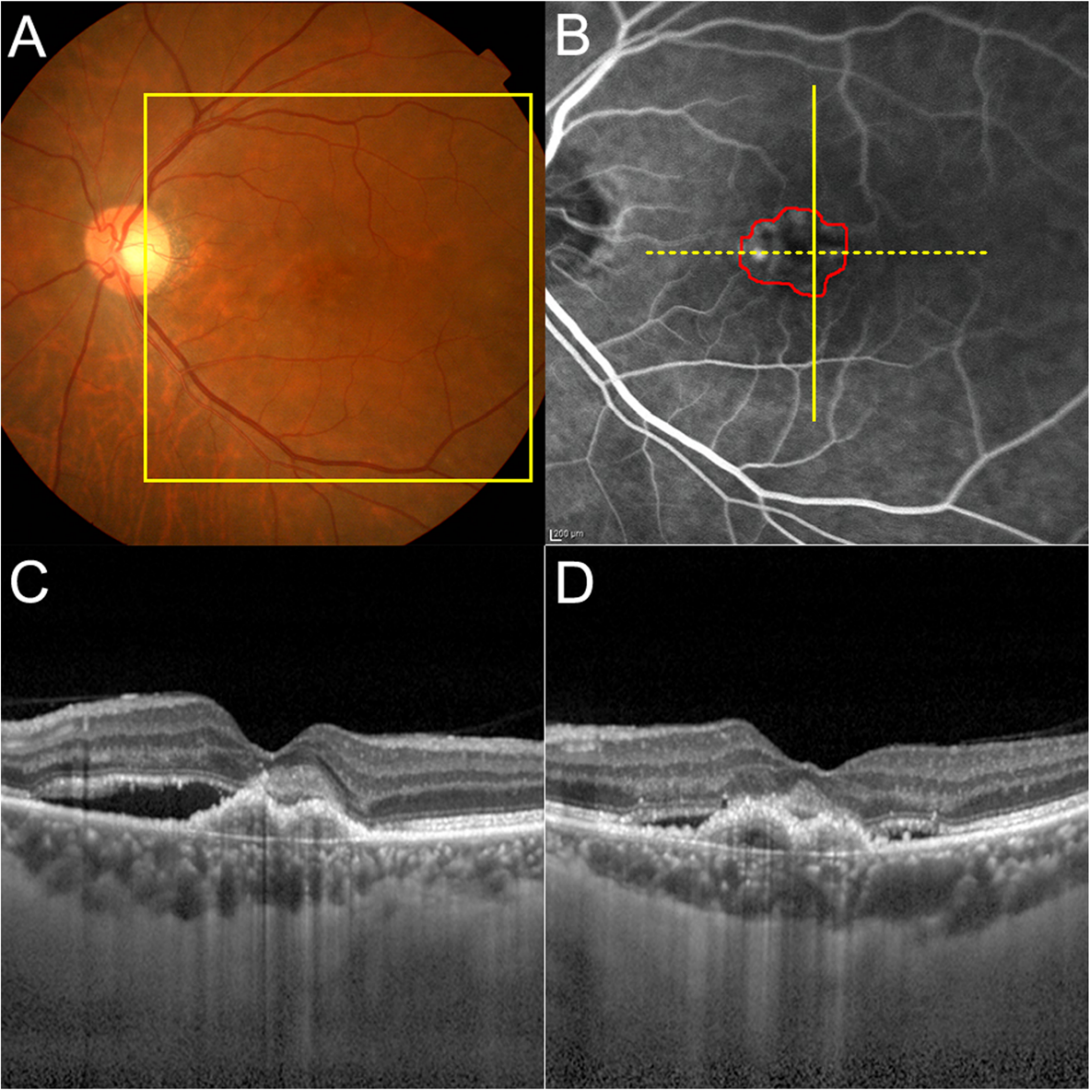
Representative images from an eye with neovascular age-related macular degeneration (nAMD). **a** Color fundus image of treatment-naïve nAMD. The square box indicates the area shown in (**b**). **b** Fluorescein angiography image. The red outline delineates an area of choroidal neovascularization (CNV) that was automatically defined by built-in software. **c, d** Optical coherence tomography images visualizing the CNV along the solid vertical line and dashed horizontal line in (**b**), respectively. The following values were measured for this eye: logMAR best corrected visual acuity = 0.22; baseline serum Diacron reactive oxygen metabolite (d-ROM) level = 339 U.CARR; serum biological antioxidant potential (BAP) = 2712 μmol/L; and CNV area = 1.72 mm^2^

**Fig. 3:**
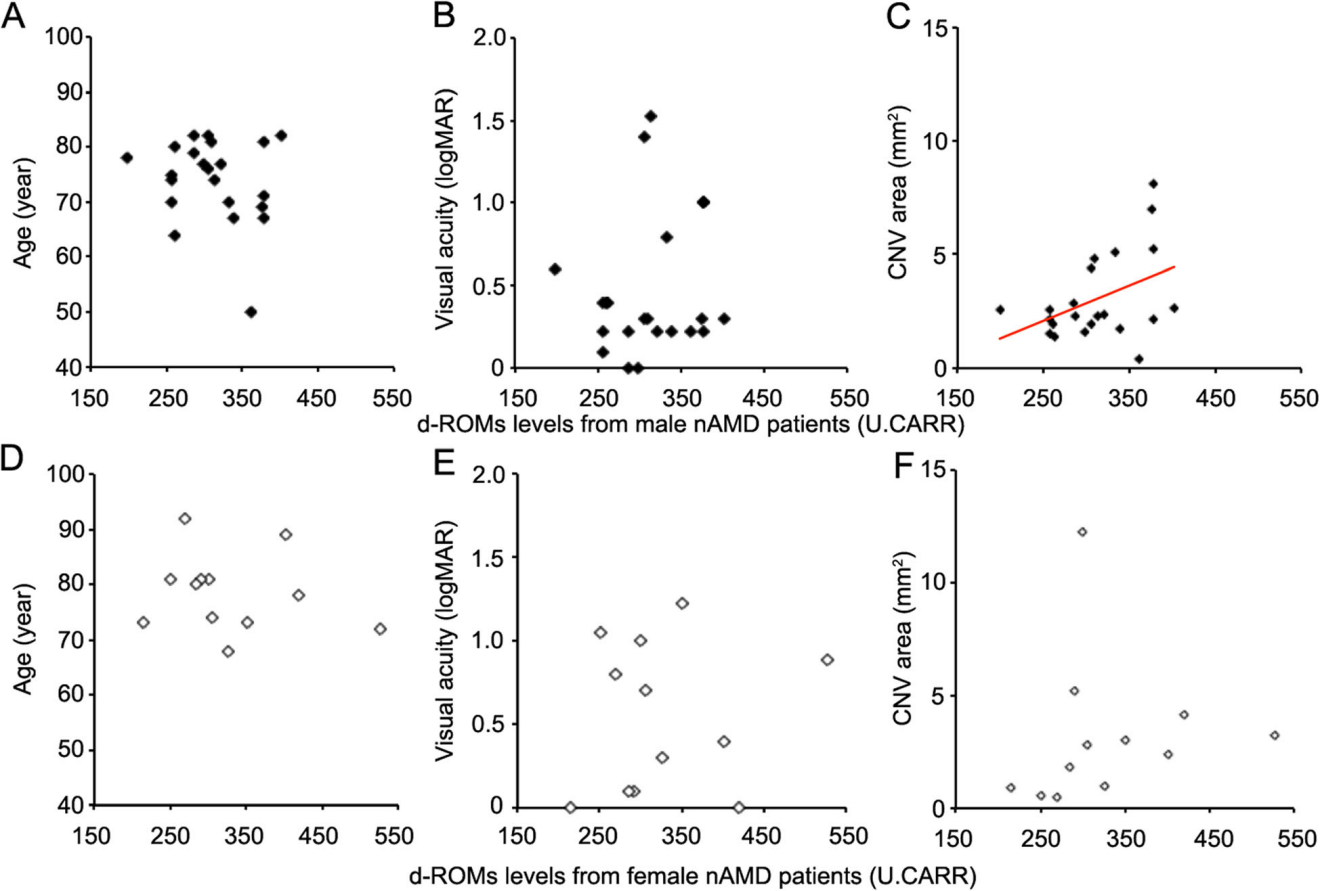
Clinical correlations with serum d-ROM levels in male (**a–c**) and female (**d–f**) neovascular age-related macular degeneration (nAMD) patients. In the male nAMD patients, d-ROM levels were significantly correlated with choroidal neovascularization (CNV) area (**c**, *P* < .05), but not with age (**a**) or visual acuity (**b**). In the female nAMD patients, d-ROM levels did not show any significant correlation with age (**d**), visual acuity (**e**), or CNV area (**f**). d-ROM = Diacron reactive oxygen metabolite

We evaluated correlations between d-ROM levels and age, logMAR BCVA, and CNV area separately for the male and female nAMD patients. A representative image of CNV in eyes with nAMD is shown in Fig. 2. There was a significant correlation between d-ROMs levels and CNV area in the male nAMD patients (*r* = .42, *P* = .05; Fig. 3c), but not in the female nAMD patients (*P* = .70; Fig. 3f). In addition, we did not find any significant correlations between d-ROMs levels and age (*P* = .35, .44; Fig. 3a and d) or logMAR BCVA (*P* = .53, .59; Fig. 3b and e) in either sex.

### Correlations between serum BAP levels and nAMD patient characteristics

**Fig. 4:**
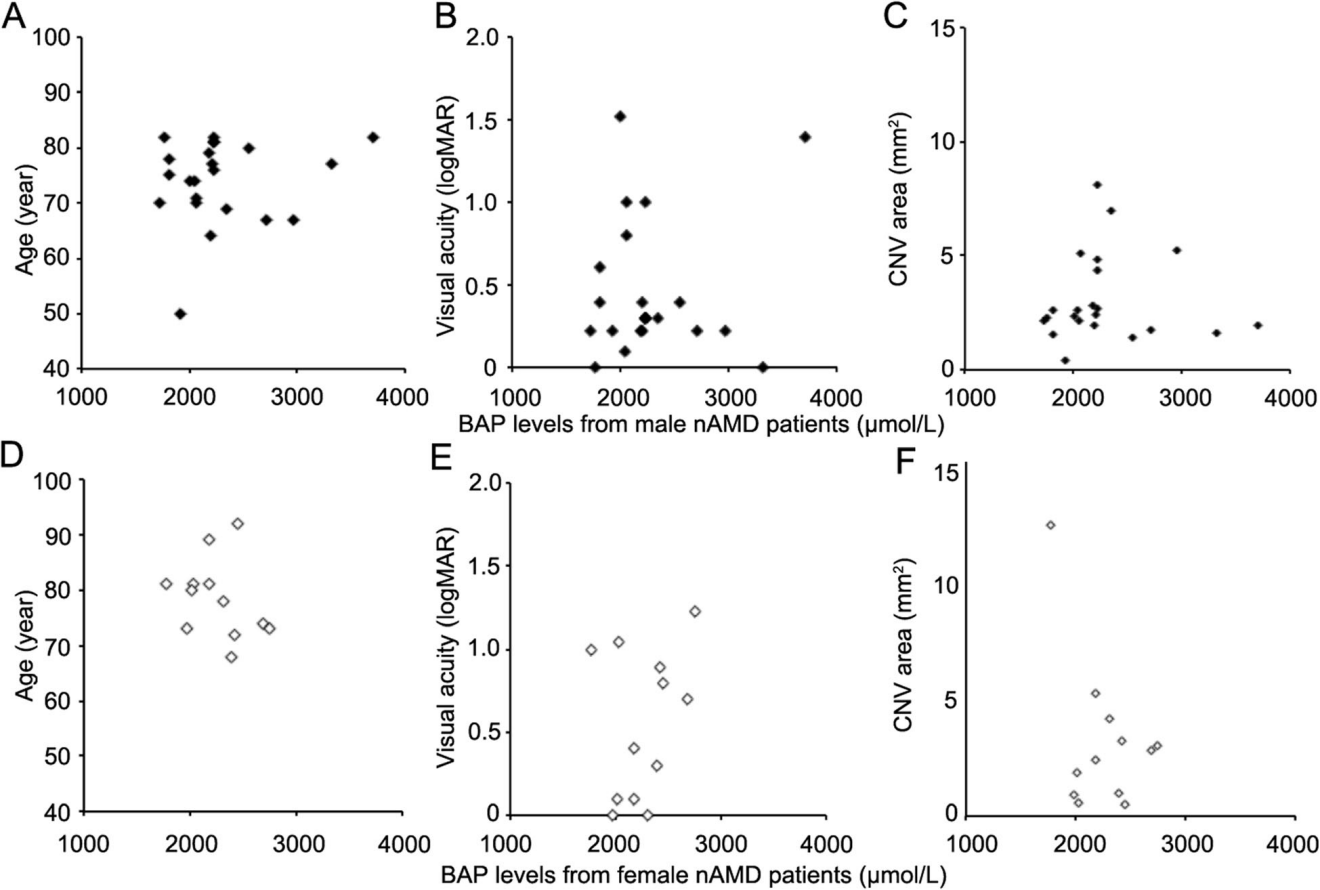
Clinical correlations with serum BAP levels in male (**a–c**) and female (**d–f**) neovascular age-related macular degeneration (nAMD) patients. No significant correlation between BAP levels and age, visual acuity, or CNV area were observed in either sex. BAP = biological antioxidant potential

We evaluated the correlations between BAP levels and age, logMAR BCVA, and CNV area. We found no significant correlations between BAP levels and age (*P* = .53 and 0.20 for male and female nAMD patients, respectively; Fig. 4a and d), logMAR BCVA (*P* = .48 and .22; Fig. 4b and e), or CNV area (*P* = .96 and .25; Fig. 4c and f).

## Discussion

Various diseases involve oxidative stress, including bronchial asthma, myocardial infarction, hypertension, diabetes, and hyperlipidemia [27, 28]. Among the ocular diseases, there has been an accumulation of evidence demonstrating a significant relationship between oxidative stress and AMD, both in vitro and in vivo [10, 29, 30]. We previously demonstrated that MDA, an oxidative stress marker, is not only a marker of AMD, but is also a direct contributor to the pathogenesis of AMD [31]. In particular, we showed that serum MDA levels in patients with nAMD were significantly higher than in control subjects, and that there was a significant correlation between serum MDA levels and CNV area [12]. The results of the present study demonstrated that, for the male subjects, serum d-ROM levels were significantly higher in nAMD patients than in control subjects (Fig. 1), and d-ROM levels were significantly correlated with CNV area (Fig. 3). These results corroborate our previous findings on MDA and suggest that the d-ROMs test could be a useful indicator of nAMD in male patients.

The study had limitations and some questions remain. (1) We did not find d-ROM levels to have biological importance in female patients with nAMD. Previous studies showed that d-ROM test results were significantly higher in women than in men of the same generation, whereas BAP test results reportedly showed no difference between men and women [32]. d-ROM levels are basically higher in women than in men and, more importantly, they increase with age for both men and women [32]. Because nAMD is a disease of older people, the female nAMD patients necessarily tended to have higher d-ROM levels. (2) We did not find a significant difference in BAP values between the nAMD and control groups for either sex. BAP is defined as the potential for antioxidation. It is possible that the negative result was because the increase in oxidative stress is more important than the decrease in antioxidative potential in the pathogenesis of nAMD. (3) The present study included small numbers. Additionally, there is a difference in age and number among the women and men in the study group. Although d-ROMs were 13.5% different between nAMD and control in male, those were only 4% different in female, there is a possibility that difference in results of men and women may be caused by it. Increasing the number of patients may indicate the association of serum d-ROMs with nAMD in women as well. Although there was no statistically significant difference in age in each group, there was variability in the age of each group, and that possibly affected the results of this study. More patients’ studies are required to confirm our findings. (4) Serum d-ROM and BAP values are affected by various diseases, diet, anti-oxidant supplements, and smoking etc. In this study, patients with diabetes, dyslipidemia, hypertension, cerebrovascular disorders, cardiovascular disorders, and cancer, as well as those under extreme stress, were also excluded, but we did not consider diet and other factors. Therefore, it is possible that oxidative stress increases due to other factors, affecting serum d-ROM and BAP values and further analysis will be required by evaluating confounding factors accurately. There is possibility that the other parameter measured in the regular blood test is associated with the d-ROM levels, and regular blood test is required for the further study for evaluating the importance of d-ROM in AMD. (5) We have reported in the past that MDA is directly involved in increasing CNV size [12]. In this study, it was found that serum d-ROM values and CNV size correlated significantly in men. However, it is unknown whether d-ROMs increased by oxidative stress is directly involved in the increase in CNV size as with MDA. These issues will be discussed in the future. (6) We previously reported that MDA value could be affected by anti-oxidant supplements. However, in this study, we did not examine the effects of anti-oxidant supplements on d-ROMs and BAP values. Further examination will be beneficial to clarify the relation between anti-oxidant supplements and d-ROMs and BAP values. (7) This study did not examine the correlation between d-ROMs and AMD activity, and there is a possibility that d-ROMs values could affect nAMD activity. Investigation of this relationship in the future might be beneficial to control nAMD activity.

The fact that the controls were significantly younger and that the confounders such as smoking (pack-years) and history of antioxidant supplementation was not accounted for in this study and that made the value of our study inherently weak. However, the fact that d-ROMs were higher in men with nAMD gives future directions of our study.

## Conclusions

In summary, the results of this study suggest that measuring serum d-ROM levels in male patients could be used as an indicator of CNV severity in nAMD.

## Supplementary information

**Additional file 1.** Raw data of patient/subject characteristics in this study. d-ROM = diacron reactive oxygen metabolite. BAP = biological antioxidant potential. CNV = choroidal neovascularization. logMAR = logarithm of the minimum angle of resolution. BCVA = best corrected visual acuity.

## Data Availability

All data generated during this study is included in this Additional file 1.

## Abbreviations

AMD: Age-related macular degeneration
BAP: Biological antioxidant potential
BCVA: Best-corrected visual acuity
CNV: Choroidal neovascularization
d-ROMs: Diacron reactive oxygen metabolites
FA: Fluorescein angiography
GA: Geographic atrophy
logMAR: Logarithm of the minimum angle of resolution
MDA: Malondialdehyde
ROM: Reactive oxygen metabolites
SEM: Standard error of the mean
VEGF: Vascular endothelial growth factor
