# Supplementary material for: Diacron reactive oxygen metabolites and biological antioxidant potential tests for patients with age-related macular degeneration
Source: BMC Ophthalmol. 2020 Feb 18;20:56. doi: 10.1186/s12886-020-01334-y (PMC7027115; doi:10.1186/s12886-020-01334-y)
Supplement: Supplementary file 1 — Additional file 1. Raw data of patient/subject characteristics in this study. d-ROM = diacron reactive oxygen metabolite. BAP = biological antioxidant potential. CNV = choroidal neovascularization. logMAR = logarithm of the minimum angle of resolution. BCVA = best corrected visual acuity. [file 12886_2020_1334_MOESM1_ESM.pdf]

Additional file 1.

Raw data of patient/subject characteristics in this study. d-ROM = diacron reactive oxygen metabolite. BAP = biological antioxidant potential. CNV = choroidal neovascularization. logMAR = logarithm of the minimum angle of resolution. BCVA = best corrected visual acuity.

	sex	d-ROMs (U.CARR)	BAP (μmol/L)	CNV size (mm^2)	smoking	logMAR BCVA
patient1	female	300	1772	12.2788	no	1
patient2	female	326	2390	0.9704	no	0.301029996
patient3	female	402	2183	2.3644	no	0.397940009
patient4	female	420	2313	4.1048	no	0
patient5	female	291	2179	5.2216	no	0.096910013
patient6	female	527	2417	3.2032	no	0.886056648
patient7	female	306	2682	2.8024	no	0.698970004
patient8	female	269	2445	0.478	no	0.795880017
patient9	female	251	2031	0.576	no	1.045757491
patient10	female	285	2012	1.8316	no	0.096910013
patient11	female	351	2749	2.9908	yes	1.22184875
patient12	female	215	1978	0.9188	no	0
patient13	male	376	2351	7.0004	yes	0.301029996
patient14	male	313	2006	2.328	no	1.522878745
patient15	male	402	2224	2.6692	yes	0.301029996
patient16	male	339	2712	1.7188	no	0.22184875
patient17	male	286	2187	2.828	no	0.22184875
patient18	male	298	3321	1.59	yes	0
patient19	male	262	2551	1.384	no	0.397940009
patient20	male	306	3705	1.9792	no	1.397940009
patient21	male	378	2963	5.2112	no	0.22184875
patient22	male	306	2227	4.3816	yes	0.301029996
patient23	male	257	2045	2.5988	yes	0.096910013
patient24	male	287	1764	2.2824	no	0
patient25	male	378	2223	8.0836	no	1
patient26	male	310	2223	4.8284	no	0.301029996
patient27	male	257	2060	2.1316	no	1
patient28	male	378	2199	1.9244	no	0.397940009
patient29	male	260	1817	2.6004	no	0.602059991

patient30	male	199	1808	1.544	no	0.397940009
patient31	male	257	1922	0.4256	no	0.22184875
patient32	male	362	2062	5.13	no	0.795880017
patient33	male	333	1724	2.1268	no	0.22184875
patient34	male	321	2208	2.3912	no	0.22184875
control1	female	361	2300		no	0.301029996
control2	female	331	2129		no	-0.11394335
control3	female	323	2564		no	0
control4	female	381	2397		no	0.096910013
control5	female	273	2528		yes	0.698970004
control6	female	391	2410		no	0.522878745
control7	female	302	2452		no	0.301029996
control8	female	341	2099		no	0
control9	female	377	2234		no	0.301029996
control10	female	345	2237		no	0
control11	male	225	2544		no	0.301029996
control12	male	231	1998		yes	0
control13	male	268	2142		no	0
control14	male	250	2125		no	0.602059991
control15	male	237	2265		no	1.22184875
control16	male	258	1602		no	-0.11394335
control17	male	286	2162		no	0
control18	male	328	2343		no	-0.11394335
control19	male	303	2021		no	0.096910013
control20	male	365	2155		yes	0.301029996
